# Robot-assisted thoracoscopic lobectomy for severe incomplete interlober fissure

**DOI:** 10.1093/jscr/rjab336

**Published:** 2021-08-14

**Authors:** Mikio Okazaki, Ken Suzawa, Kazuhiko Shien, Kentaroh Miyoshi, Shinji Otani, Hiromasa Yamamoto, Seiichiro Sugimoto, Masaomi Yamane, Shinichi Toyooka

**Affiliations:** Department of Thoracic, Breast and Endocrinological Surgery, Okayama University Graduate School of Medicine, Okayama, Japan; Department of Thoracic, Breast and Endocrinological Surgery, Okayama University Graduate School of Medicine, Okayama, Japan; Department of Thoracic, Breast and Endocrinological Surgery, Okayama University Graduate School of Medicine, Okayama, Japan; Department of Thoracic, Breast and Endocrinological Surgery, Okayama University Graduate School of Medicine, Okayama, Japan; Department of Thoracic, Breast and Endocrinological Surgery, Okayama University Graduate School of Medicine, Okayama, Japan; Department of Thoracic, Breast and Endocrinological Surgery, Okayama University Graduate School of Medicine, Okayama, Japan; Department of Thoracic, Breast and Endocrinological Surgery, Okayama University Graduate School of Medicine, Okayama, Japan; Department of Thoracic, Breast and Endocrinological Surgery, Okayama University Graduate School of Medicine, Okayama, Japan; Department of Thoracic, Breast and Endocrinological Surgery, Okayama University Graduate School of Medicine, Okayama, Japan

## Abstract

An incomplete interlobar fissure makes thoracoscopic lobectomy difficult and is predictive of morbidity after thoracoscopic lobectomy. This report demonstrates the robot-assisted thoracoscopic (RATS) lobectomy technique for patients with severe incomplete interlobar fissures. A fissureless approach was chosen for pulmonary resection. Near-infrared fluorescence imaging with intravenous indocyanine green (ICG) was used to detect the interlobar line after transection of the bronchus, pulmonary artery and vein. Interlobar fissure was identified and divided by robotic staplers. This combined technique using ICG and fissureless lobectomy made RATS lobectomy safe for patients with severe incomplete interlobar fissures.

## INTRODUCTION

Incomplete interlobar fissures increase the surgical difficulty of video-assisted thoracoscopic surgery (VATS) lobectomy. Furthermore, the degree of pulmonary fissure completeness is a categorical predictor of both major and minor morbidity after VATS lobectomy [1]. Therefore, the robot-assisted thoracoscopic (RATS) approach is sometimes avoided for patients with incomplete interlobar fissure, indicated for lobectomy. This study demonstrates a useful RATS lobectomy technique in patients with severe incomplete interlobar fissures.

## TECHNIQUE

**Figure 1:**
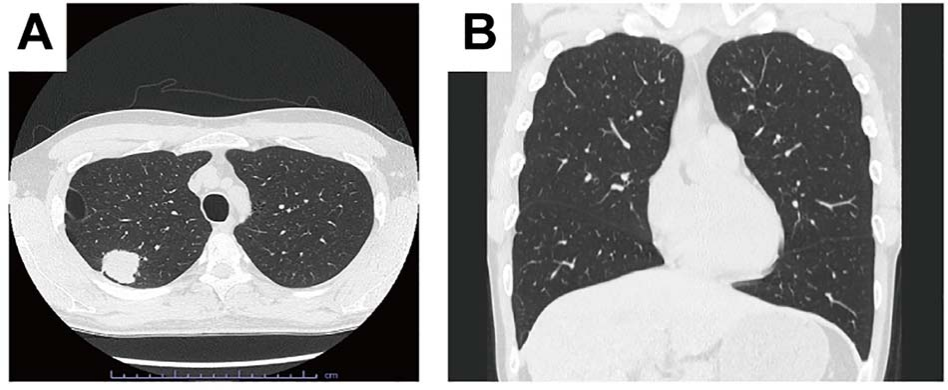
Computed tomography showed a 33 mm mass in the right upper lobe. A. No interlobar fissure between the right upper lobe and middle lobe

This is the case of a 38-year-old man with cStage IB adenocarcinoma. Computed tomography showed a 33-mm mass in the right upper lobe (Fig. 1A), and no interlobar fissure between the right upper and middle lobes (ho. 1B).

**Figure 2:**
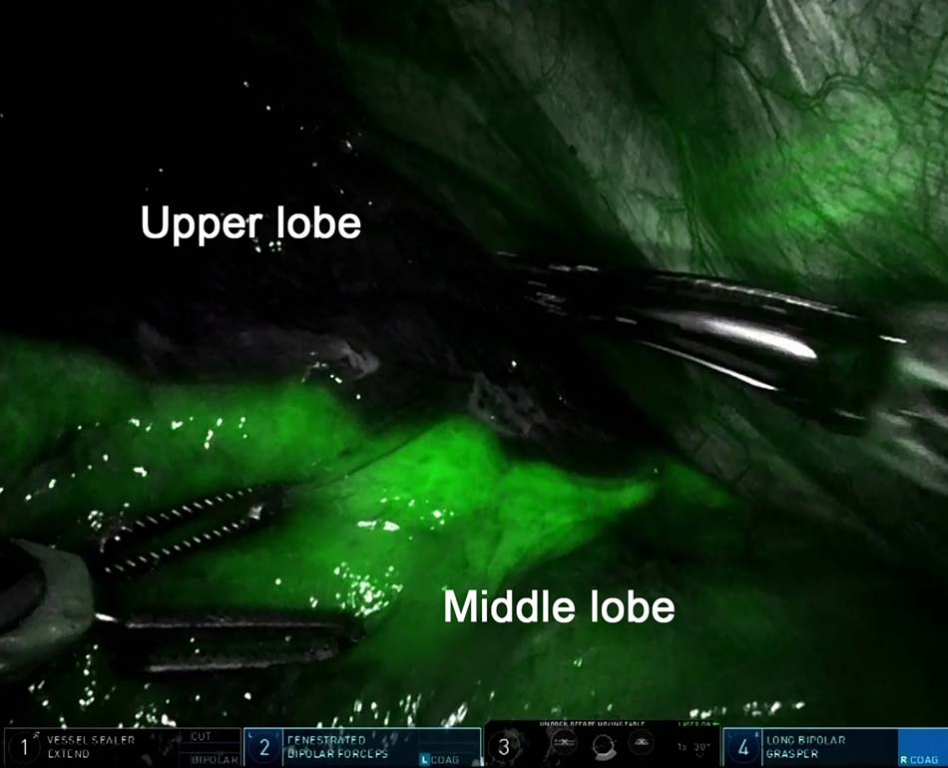
Intravenous indocyanine green delineation of the interlobar border between the right upper and middle lobe.

RATS right upper lobectomy with ND2a-2 was performed via four port incisions and one assist incision. One 12-mm robotic port was placed in the eighth intercostal space on the anterior axillary line, and three 8-mm robotic ports were placed in the eighth intercostal space. The assist incision was made in the fourth intercostal space on the anterior axillary line. The Da Vinci Xi surgical system (Intuitive Surgery, Sunnyvale, CA, USA) was docked. A vessel sealer extended, fenestrated bipolar forceps and long bipolar grasper were inserted. Then the upper bronchus, V1 + 2 + 3, and A1 + 2 + 3 were exposed and transected by a robotic stapler. To identify the minor fissure, indocyanine green (ICG) (7.5 mg) was injected through the peripheral venous catheter, followed by a 10-ml flush of sterile normal saline. After injecting, the surgical field was visualized with the integrated fluorescence imaging capability of the Firefly Fluorescence Imaging camera (Intuitive Surgical) (Fig. 2). The interlobar border was then identified as the line separating the dark lung parenchyma from the green lung parenchyma. The border was marked on the visceral pleura using a long bipolar grasper and divided by robotic staples. The resected lobe was extracted after mediastinal node dissection. The total operation time was 2 h 38 min, and the console time was 1 h 34 min. There was minimal blood loss. The operation was completed without complications, and the postoperative course was uneventful.

**Video:**
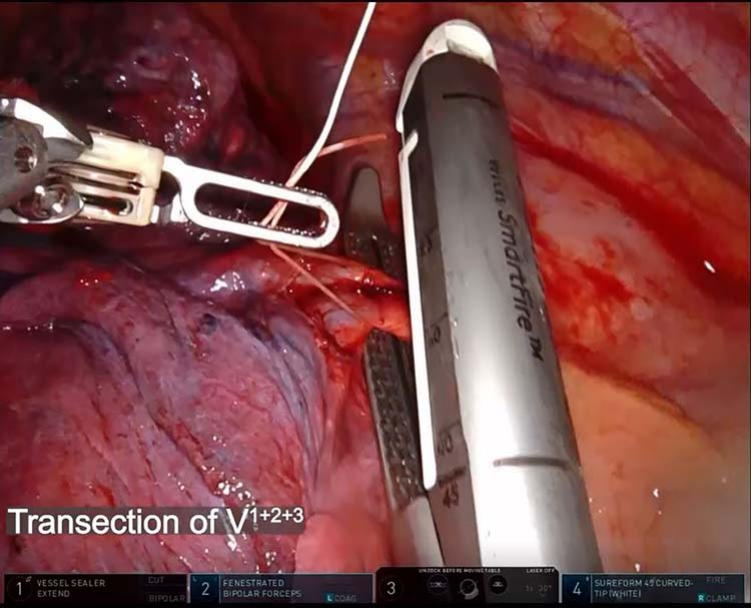
Indocyanine green was intravenously injected after transection of the pulmonary artery, vein and bronchus by robotic staplers. The interlobar fissure was identified by near-infrared fluorescence imaging camera and divided by robotic staplers.

## DISCUSSION

To the best of our knowledge, this was the first report on RATS lobectomy using the combined ICG and fissureless technique for patients with severe incomplete interlobar fissure. We used this technique in three cases, including the present case. All operations were completed without complications, and the postoperative courses were uneventful.

Fissureless VATS lobectomy has been reported as a superior alternative to conventional lobectomy in preventing prolonged air leak, and shortening hospital stay and chest tube duration [2, 3]. Although the efficacy of the fissureless technique in RATS lobectomy has not been reported, this technique is also useful in RATS lobectomy for patients with moderate and severe incomplete fissures. In addition, near-infrared fluorescence mapping with ICG was recently reported as a helpful advancement to identify the intersegmental plane in minimally invasive segmentectomy [4, 5]. Da Vinci Xi is equipped with this technology, and we applied this technique to identify the interlobar fissure in RATS lobectomy.

Compared to VATS, RATS was associated with a lower conversion rate in meta-analyses [6]. However, RATS required a safer procedure than VATS because the conversion to open thoracotomy from RATS takes longer than VATS in emergency cases. The technique discussed in this study made RATS lobectomy for patients with severe incomplete interlobar fissure safer and easier to perform. Therefore, it can contribute to reducing the conversion rate for incomplete interlobar fissure cases.

## Supplementary Material

RATS_incomplete_fissure_rjab336
